# A Rare Case of Seronegative Lupus Nephritis Presenting as Immune Complex Diffuse Glomerulonephritis

**DOI:** 10.7759/cureus.86406

**Published:** 2025-06-20

**Authors:** Eugene K Yeboah, Isha Puri, Mary Mallappallil, Sonalika Agarwal, Crystal V Delp, Fnu Suraj, Subodh Saggi, Muhammad Azhar

**Affiliations:** 1 Internal Medicine, State University of New York Downstate Medical Center, Brooklyn, USA; 2 Internal Medicine, New York City Health and Hospitals Corporation (HHC) Kings County Hospital Center, Brooklyn, USA; 3 Nephrology, State University of New York Downstate Medical Center, Brooklyn, USA; 4 Nephrology, New York City Health and Hospitals Corporation (HHC) Kings County Hospital Center, Brooklyn, USA

**Keywords:** full house nephropathy sle, full-house pattern immunofluorescence, lupus nephritis, seronegative lupus, seronegative lupus nephritis

## Abstract

Lupus nephritis (LN) is a well-characterized renal manifestation of systemic lupus erythematosus (SLE), typically supported by serologic markers such as antinuclear antibody (ANA) and anti-double stranded deoxyribonucleic acid (anti-dsDNA). However, seronegative presentations remain rare and diagnostically elusive, particularly when renal involvement precedes other systemic features. We report a case of a 31-year-old male with type 1 diabetes who presented with progressive anasarca, dyspnea, and a diffuse non-blanching rash. Laboratory evaluation revealed nephrotic-range proteinuria, hypoalbuminemia, and elevated serum creatinine. Despite strong clinical suspicion, comprehensive autoimmune serologies, including ANA, anti-dsDNA, extractable nuclear antigen (ENA) panel, and anti-neutrophil cytoplasmic antibodies (ANCA), were negative. Renal biopsy demonstrated diffuse proliferative glomerulonephritis with a full house immunofluorescence pattern, including granular capillary wall staining for immunoglobulin G, A, and M (IgG, IgA, and IgM), complement 1q (C1q), and complement 3 (C3). These findings, in the absence of secondary causes such as infection, paraproteinemia, or cryoglobulinemia, supported a diagnosis of seronegative lupus nephritis. The patient was initiated on corticosteroids and mycophenolate mofetil, with resolution of edema and stabilization of renal function. This case highlights the diagnostic significance of renal biopsy in seronegative LN. The presence of a full house immune complex deposition pattern should prompt consideration of LN regardless of serologic status. While rare, seronegative LN underscores the limitations of relying solely on serologies for diagnosis and reinforces the central role of histopathology in complex glomerular disease. In patients presenting with immune complex-mediated glomerulonephritis and absent serologic markers, seronegative lupus nephritis should remain on the differential. Renal biopsy not only establishes diagnosis but also guides timely immunosuppressive therapy critical to preserving renal function.

## Introduction

Systemic lupus erythematosus (SLE) is a chronic, multisystemic, autoimmune connective tissue disorder with diverse clinical presentations [1]. The diagnosis of SLE can be made based on clinical and laboratory parameters using the American College of Rheumatology (ACR), which requires 4 out of 11 clinical criteria, or Systemic Lupus International Collaborating Clinics (SLICC) criteria, which requires 4 of 17 criteria, including at least one clinical criterion and one immunologic criterion or biopsy-proven lupus nephritis [2]. The majority of SLE cases are seropositive, which depends on positive tests for antinuclear antibody (ANA), lupus erythematosus (LE) cells, and antibody to deoxyribonucleic acid (DNA), but seronegative SLE cases also rarely can present with complications [3]. ANA testing is positive for virtually all patients with SLE at some time in the course of their disease. However, in early diseases, up to 6% of patients may have a negative ANA by immunofluorescence [4]. We present a rare case of seronegative lupus nephritis presenting as immune complex diffuse glomerulonephritis.

## Case presentation

A 31-year-old male presented with generalized body swelling and shortness of breath for three weeks. The patient has a medical history of type one diabetes, diabetic retinopathy, and latent tuberculosis (never treated). He presented with shortness of breath and progressive body swelling. He initially noticed his feet were swollen about 3 to 4 months prior. He reported to a different emergency department and was told that he may have a kidney or heart problem. He was prescribed some medications and discharged home to follow up. He reported improvement in his symptoms, including lower extremity swelling, several weeks later. However, the swelling returned about three weeks prior to reporting to us. The swelling was more severe than before and involved both his upper extremities and lower back. This time the swelling was associated with shortness of breath and difficulty walking due to bilateral knee pain. He also noticed that his face was swollen, which prompted him to come to the emergency room. He also had a three-week history of diffuse rash involving his back, torso, and extremities but sparing his face. He smoked marijuana, was a construction worker, and had a history of intermittent cocaine use. He was sexually active with a single female partner and had no family history of kidney disease, but both his parents had diabetes. His home medications included Losartan 40 mg daily, Carvedilol 3.125 mg twice daily, Cetirizine 10 mg daily, Diphenhydramine 25 mg daily, Furosemide 20 mg twice daily, Aspart Insulin 5 units three times daily, Glargine Insulin 25 units nightly, and 1% Terbinafine cream twice daily.

His initial vitals were a temperature of 36.5°C, a heart rate of 95 bpm, a respiratory rate of 18 cycles per minute, oxygen saturation on room air of 97%, blood pressure of 172/87, and a body mass index of 30.79 kg/m². The patient looked ill and had anasarca. He had bilateral chemosis. He was not in respiratory distress but had fine basal crackles and rales on lung auscultation. He had a maculopapular rash on his back. He had bilateral lower extremities pitting pedal edema with an overlying erythematous patch. There were obvious non-blanching erythematous macules and patches on bilateral shins. There were erythematous, ill-defined macules in bilateral arms. The scalp had three excoriated papules. The groin, oral mucosa, and ocular examination were unremarkable.

The initial workup is summarized in Table 1.

**Table 1:** Initial workup APTT: Activated Partial Thromboplastin Time, PT: Prothrombin Time, INR: International Normalized Ratio

Parameter	Patient Values	Reference Range
Comprehensive Metabolic Panel		
Sodium	138 mmol/L	136-145 mmol/L
Potassium	5.3 mmol/L	3.5-5.1 mmol/L
Calcium	8.0 mg/dL	8.2-10.0 mg/dL
Chloride	109 mmol/L	98-107 mmol/L
Creatinine	2.67 mg/dL	0.7-1.3 mg/dL
Blood Urea Nitrogen	67.0 mg/dL	7-25 mg/dL
Carbon dioxide	20 mmol/L	21-31 mmol/L
Glucose	215 mg/dL	70-99 mg/dL
Anion Gap	9 mmol/L	10-20 mmol/L
Beta hydroxybutyrate	0.10 mmol/L	0,10-0.40mmol/L
Estimated Glomerular Filtration Rate	38.1ml/min/1.73m²	>60 ml/min/1.73m²
Liver Function Test		
Total Bilirubin	<0.1 mg/dL	0.3-1.0 mg/dL
Albumin	1.8 g/dL	3.5-5.7 g/dL
Total Protein	5.5 g/dL	6.0-8.3 g/dL
Aspartate aminotransferase	20 U/L	13-39 U/L
Alanine aminotransferase	11 U/L	7-52 U/L
Alkaline phosphatase	166 U/L	34-104 U/L
Glycated Hemoglobin (Hb A1C)	7.7%	<4.2-5.6%
Complete Blood Count		
Hemoglobin	11.90 g/dL	14.0-18.0 g/dL
White Blood Count	12.4k/μL	3.5-10.8 k/μL
Platelet	304k/μL	130-400k/μL
Hematocrit	39.6%	42.0-52.0%
Urinalysis		
Appearance	Clear	Clear
pH	6.0	5.0-8.0
Specific gravity	1.018	1.005-1.030
Urine glucose	500mg/dL	Negative mg/dL
Urine Blood	Small	Negative
Red blood cells urine	10.2/HPF	0.0-5.0/HPF
Urine Protein	300mg/dL	Negative mg/dL
Urine nitrite	Negative	Negative
Leucocyte esterase	Negative	Negative
White Blood Cells (Urine)	4.3/HPF	0-5/HPF
Urine Cast	20/LPF	0-2/LPF
Clotting Profile		
APTT	42 seconds	25-37 seconds
PT	12.2 seconds	9.4-12.5 seconds
INR	1.1	0.8-1.2
Brain Natriuretic Peptide	1754 pg/mL	1-25pg/mL
High sensitivity Troponin	17ng/L	0-22ng/L

The patient's presentation, including his history, physical examination, low albumin, proteinuria, hematuria, and impaired renal function, prompted further work, with our top differential diagnoses being nephrotic syndrome, nephritic syndrome, and diabetic nephropathy. We also considered heart failure because he had diabetes, hypertension, anasarca, and elevated brain natriuretic peptide. The patient was prescribed intravenous furosemide, metolazone, and intravenous albumin for diuresis while admitted for workup. While on admission, his blood pressure was controlled with amlodipine 10 mg daily, carvedilol 12.5 mg twice daily, losartan 50 mg daily, empagliflozin 10 mg daily, and adjustment doses of furosemide and metolazone. His blood glucose was monitored and controlled with incremental doses of insulin. Concerning his skin findings, there was a concern for adult-onset Henoch-Schoenlein purpura (HSP) and other vasculitic syndromes with anti-neutrophil cytoplasmic antibody (ANCA) vasculitis versus edema-induced skin changes. Dermatologists recommended a shave biopsy and steroids while waiting for biopsy results. We considered lupus nephritis (LN), ANCA vasculitis, and immunoglobulin A (IgA) vasculitis due to the skin findings together with impaired renal function. The patient was managed by a multidisciplinary team including internal medicine physicians, nephrologists, rheumatologists, dermatologists, and cardiologists.

**Figure 1:**
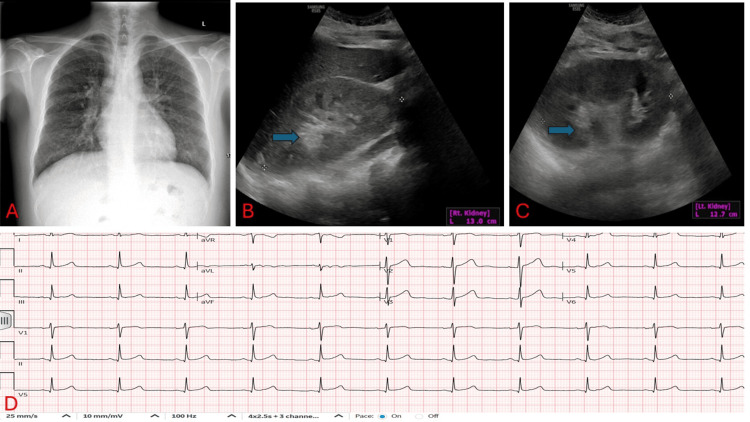
The patient’s electrocardiogram, chest X-ray, and kidney ultrasound A: Chest X-ray negative for consolidation and pleural effusion with normal cardiac silhouette B: Right Kidney: measures 13 cm with no evidence of hydronephrosis or calculi. However, there is an increased cortical echogenicity (blue arrow) C: Left Kidney: measures 12.7 cm with no evidence of hydronephrosis or calculi. However, there is an increased cortical echogenicity (blue arrow) D: Electrocardiogram showed normal sinus rhythm with a rate of 65 bpm, PR interval of 138 ms, QRS duration of 74 ms, and QTC of 409 ms

The patient’s electrocardiogram, chest X-ray, and kidney ultrasound are displayed in Figure 1.

Subsequent lab work done is summarized in Table 2.

**Table 2:** Subsequent lab workup IgM: Immunoglobulin M, IgG: Immunoglobulin G, IgA: Immunoglobulin A, TB: Tuberculosis, Anti: Antibody

Parameter	Patient value	Reference range
Lipid panel		
Total cholesterol	220 mg/dL	0-199mg/dL
Triglyceride	287 mg/dL	0-150 mg/dL
Low density lipoprotein cholesterol	135 mg/dL	60-129 mg/dL
High density lipoprotein cholesterol	33 mg/dL	>55mg/dL
Further urine examination		
Random urine sodium	31mmol/L	20-100mmol/L
Random urine potassium	33.7mmol/L	20-125mmol/L
Random urine chloride	27mmol/L	30-260mmol/L
Random urine urea	461.6mg/dL	801.0-1666.0mg/dL
Random urine creatinine	92.69 mg/dL	39.00-259.00 mg/dL
Random urine protein	286mg/dL	0-12mg/dL
Protein/Creatinine Ratio	3,086 mg/g	22-128 mg/g
Iron studies		
Ferritin	133 ng/mL	30.00-400.00 ng/mL
Iron	28 ug/dL	59-158 ug/dL
Total Iron Binding Capacity (TIBC)	98 ug/dL	250-410ug/dL
Unsaturated Iron Binding Capacity (UIBC)	70 ug/dL	112-410ug/dL
Glomerulopathy work up		
Complement (C3) levels	127mg/dL	86-184mg/dl
Complement (C4) levels	29mg/dL	20-58mg/dl
Serum Immunofixation	No monoclonal band	No monoclonal band
Total serum protein electrophoresis	Hypogammaglobulinemia and hypoalbuminemia	Normal
Kappa Free Light Chain	12.80 mg/dL	0.33-1.94mg/dL
Lambda Free Light Chain	8.30 mg/dL	0.57-2.63 mg/dL
Kappa Lambda Free Light Chain Ratio	1.54	0.26-1.65
Infectious work up		
Urine Chlamydia Polymerase Chain Reaction (PCR)	Negative	Negative
Venereal disease research Laboratory (VDRL)	Negative	Negative
Human immunodeficiency virus 1/2 Antigen/Antibodies	Negative	Negative
Hepatitis C virus antibody	Non-reactive	Non-reactive
Hepatitis B surface antigen	Non-reactive	Non-reactive
Hepatitis B surface antibody	Reactive	Non-reactive
Hepatitis B core antibody	Non-reactive	Non-reactive
Hepatitis A antibody	Reactive	Non-reactive
Epstein- Barr Virus IgM	<10 U/ml	N<35.90U/ml
QuantiFERON TB Gold	Positive	Negative
Autoimmune work up		
Thyroid stimulating hormone	3.340 uIU/mL	0.270-4.200Uiu/mL
Antinuclear antibody (ANA)	Negative	<1:80
Anti-streptolysin O	64IU/mL	0-64IU/ml
Atypical Anti-neutrophil cytoplasmic antibodies (ANCA)	Negative	Negative
Cardiolipin Antibodies IgG, Enzyme immunoassay (EIA)	<16 GPL U/mL	<19.9 GPL U/mL
Cardiolipin Antibodies IgM, EIA	<1.5 MPL U/mL	<19.0 MPL U/mL
Extractable Nuclear Antigen (ENA)	<0.2 AI	<0.9AI
Histone antibodies	0.3 units	0.0-0.9 units
Perinuclear (P-ANCA) antibodies	Negative	Negative
Rheumatoid Factor	14 IU/ML	≤14 IU/mL
Anti double stranded DNA antibodies (anti-dsDNA), EIA	<1	≤ 4 IU/mL
ENA-Smith Antibodies	<0.2 AI	≤ 0.9 AI
Topoisomerase I (SCL-70) antibodies	<0.2 AI	≤ 0.9AI
Cytoplasmic (C-ANCA) antibodies	Negative	Negative
Cardiolipin antibodies IgA	<2.0 APL U/mL	≤ 19.9 APL U/mL
Myeloperoxidase antibodies	<0.2 AI	≤ 0.9 AI
Proteinase 3 antibodies, EIA	<0.2 AI	≤ 0.9 AI
SSA-52 IgG (anti-Ro)	5 AU/mL	0-40 AU/mL
SSA-60 1gG (anti-La)	0 AU/mL	0-40 AU/mL
Cryoglobulins	Negative	Negative

The patient’s chest X-ray was normal with a normal cardiac silhouette, and his echocardiogram showed a preserved ejection fraction of 65%, normal wall motion, normal ventricular and atrial sizes, normal heart valves, and normal pulmonary heart pressures. His kidney ultrasound showed enlarged kidneys with increased cortical echogenicity consistent with medical renal disease.

**Figure 2:**
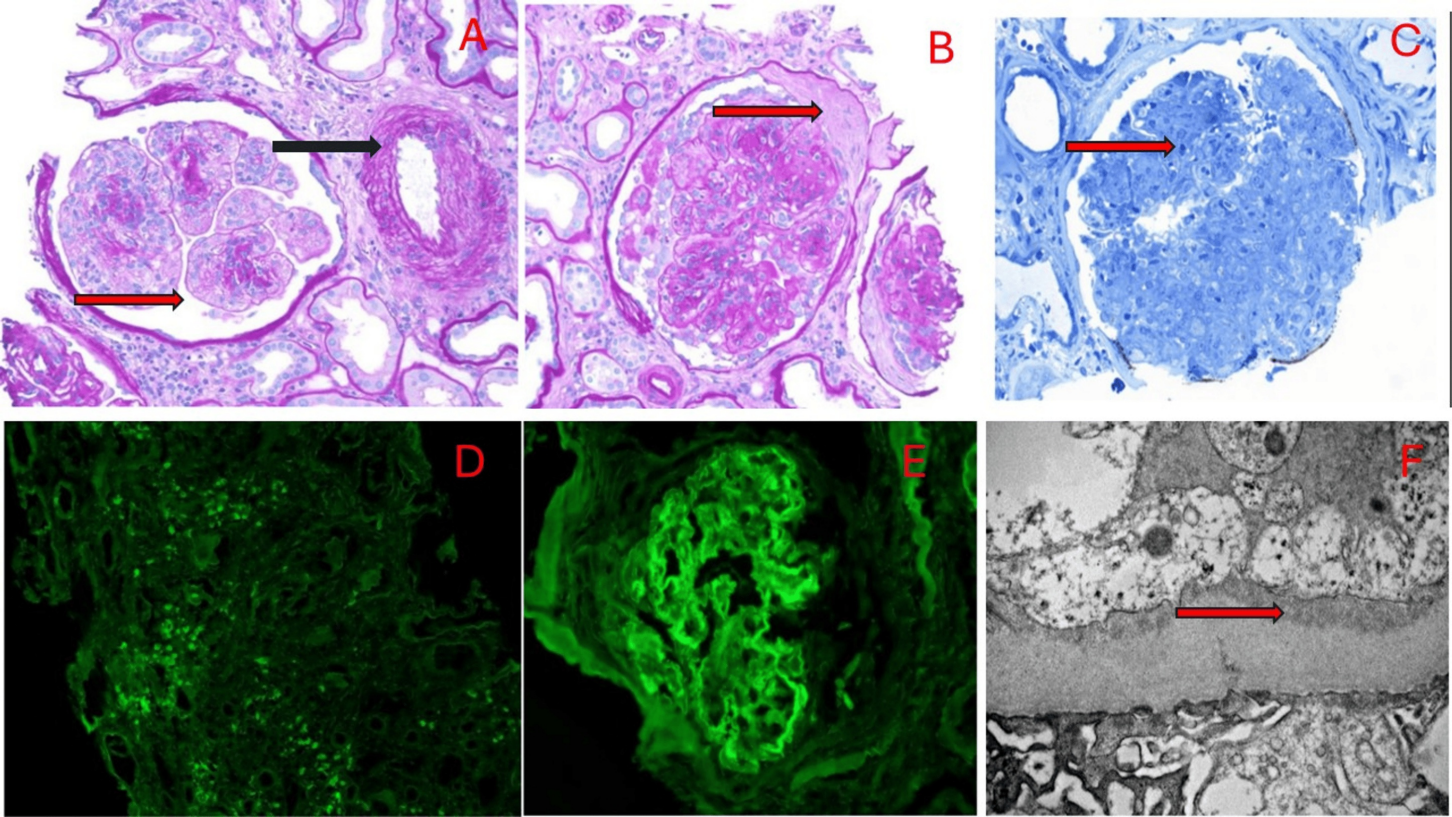
Biopsy of the patient A: Light microscopy with H&E stain showing Mesangiolysis (red arrow) and arteriosclerosis (black arrow) B: Light microscopy with H&E stain showing crescent glomerulosclerosis (red arrow) C: Light microscopy with Toluidine blue stain showing glomerulus with endocapillary hypercellularity (red arrow), basement membrane thickening, and segmental mesangial matrix expansion with segmental nodularity. D: Direct immunofluorescence showing plasma cells (IgG immunofluorescence) E: Direct immunofluorescence showing C1q immunofluorescence. The glomeruli have granular capillary walls and segmental mesangial staining with antisera specific for IgG (2+), IgA (trace), IgM (2+), C3 (2+), C1q (3+), kappa light chains (2+), and lambda light chains (2+) F: Electron microscopy showing endocapillary hypercellularity, basement membrane thickening, segmental mesangial matrix expansion with segmental nodularity, segmental tuft lumen inflammatory cells, foam cells, subendothelial immune complex type capillary wall electron-dense deposits, rare individual subepithelial immune complex type electron-dense deposits, small mesangial matrix immune complex type electron-dense deposits, and capillary wall deposits (red arrow)

The patient's renal biopsy showed immune complex diffuse glomerulonephritis with full house staining positive on immunofluorescence with trace IgA and chronic active interstitial nephritis and active tubular necrosis. It revealed severe, diffuse acute tubular epithelial cells and tubulointerstitial nephritis, immune complex-mediated diffuse proliferative glomerulonephritis with two (17%) crescents. It also showed diabetic glomerulosclerosis, Renal Pathology Society (RPS) 2010 diabetic nephropathy class III, nodular sclerosis, and Kimmelstiel-Wilson nodules. There are granular capillary walls and segmental mesangial staining for Immunoglobulin G (IgG) 2+, trace IgA, Complement 3 (C3) 2+, Complement 1q (C1q) 3+, and kappa lambda 2+. Kidney biopsy is represented in Figure 2.

Initial concern for IgA/HSP vasculitis, but trace IgA on immunofluorescence made this diagnosis unlikely. Autoimmune workup, including ANA, anti-double-stranded deoxyribonucleic acid (anti-dsDNA), ANCA panel, rheumatoid factor, infectious workup of anti-streptolysin O (ASO) negative, blood culture, Bartonella, and monoclonal gammopathy with complements, were within normal limits. The patient's skin biopsy revealed perivascular dermatitis with a differential diagnosis of drug eruption versus viral eruption. There were no features of vasculitis. ANCA vasculitis, as the patient had pauci-immune glomerulonephritis and the ANCA panel was negative. Cryoglobulin level was negative, thus low suspicion for cryoglobulinemic vasculitis. The patient’s history, physical examination, and workup, including kidney biopsy, made seronegative lupus the most probable cause of the full-house immune complex glomerulonephritis.

The patient was initially prescribed a pulse steroid of intravenous methylprednisolone 500 mg daily for three days, followed by oral prednisolone 40 mg daily. The patient's skin findings resolved, and kidney function minimally improved after starting steroid therapy; however, the kidney function remained static with creatinine persistently above 2.6 and proteinuria. He was subsequently prescribed oral mycophenolate mofetil, 500 mg twice daily for one week, and subsequently increased to 1000 mg twice daily while on prednisolone. The patient’s symptoms improved, and his kidney function returned to baseline. The patient was discharged home to continue mycophenolate mofetil 1000 mg twice daily together with a tapered dose of prednisolone and all other medications. The tapered dose of prednisolone included oral prednisolone 40 mg daily for 28 days, followed by 30 mg daily for 28 days, followed by 20 mg daily for 28 days, then 10 mg daily for 28 days, and further tapering based on response with the aim of <5 mg prednisolone per day. While the patient was on steroid therapy, he was prescribed steroid prophylaxis with oral vitamin D3 1000 units daily, calcium carbonate 1250 mg daily, famotidine 20 mg daily, and atovaquone 1500 mg daily for pneumocystis jirovecii. He was scheduled to follow up with a nephrologist, primary care physician, cardiologist, and rheumatologist. Following discharge, the patient's kidney function has been stable over several months, and they continue to follow up with a nephrologist.

Figure 2 shows the trend of kidney function: plasma creatinine (left) and urine protein creatinine ratio (right) after starting treatment.

**Figure 3:**
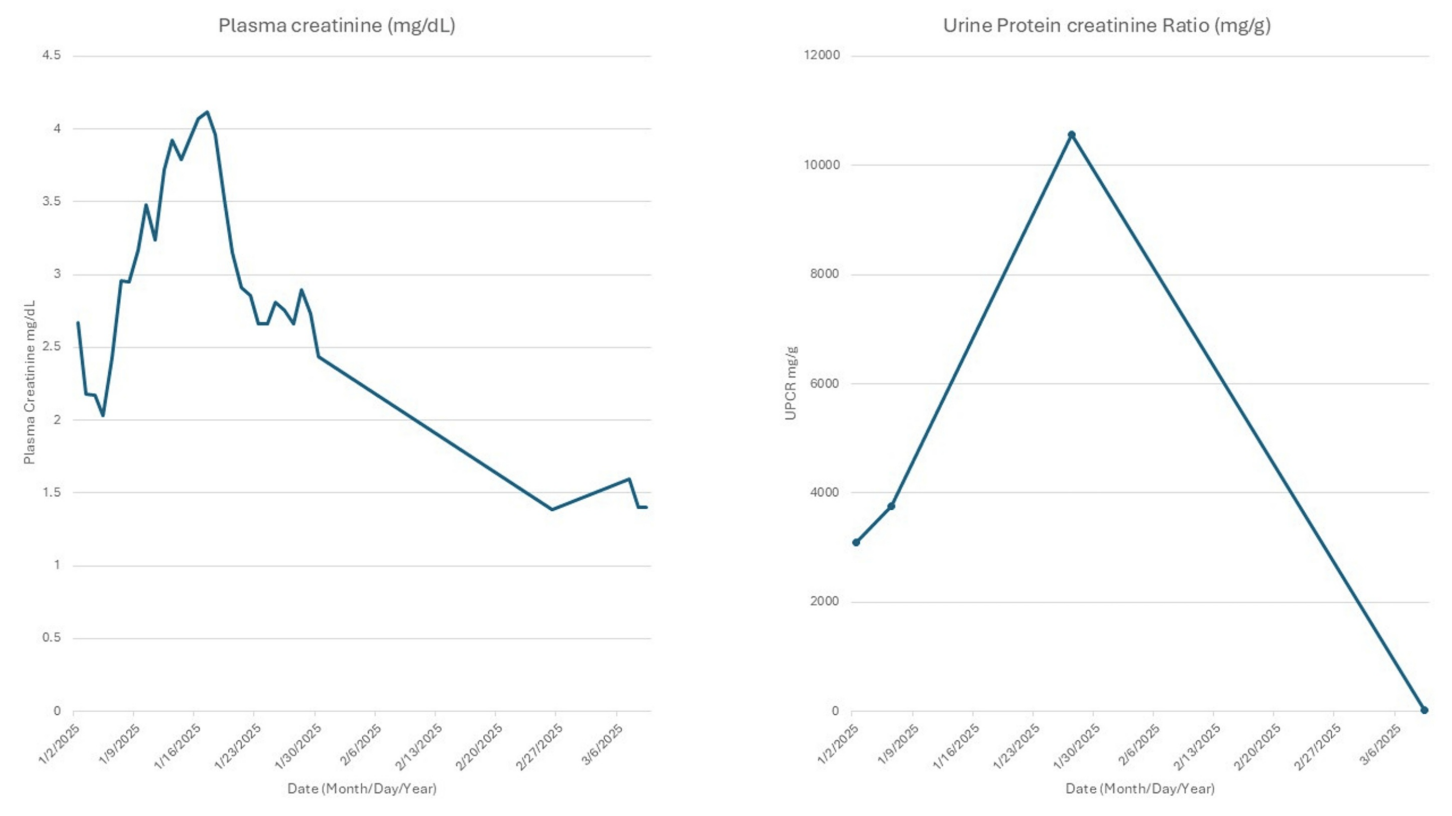
Trend of kidney function after initiating treatment

## Discussion

SLE is a multi-system autoimmune disorder with unknown etiology and presents as the deposition of pathologic autoantibodies and immune complexes into the tissues [3]. Immunological abnormalities, particularly the production of antinuclear antibodies (ANA), are a prominent feature of the disease. However, there are instances where ANA is negative, popularly known as seronegative lupus. This rare entity of lupus was first reported in 1976 [5]. In the first few reported cases of seronegative lupus, skin manifestation, especially photosensitivity, was the dominant feature [5]. According to numerous studies, seronegative negative SLE is an infrequent entity of lupus, with an incidence of <4% among all SLE patients [6]. SLE has high mortality and morbidity, with 5-year and 10-year survival rates of about 90-95% and 70-85%, respectively [7]. These alarming figures are the reasons to not solely rely on ANA in making diagnoses, especially if there is a high index of suspicion for lupus, as early treatment can change the course of the disease.

Kidney involvement is clinically present in about 50% of SLE patients and is a significant cause of morbidity and mortality [8]. A significant portion of lupus nephritis (LN) patients, about 10%, will eventually advance to end-stage kidney disease [8-12]. Almost all SLE patients will have objective evidence of renal pathology at some point in the disease course, usually initially demonstrated as an abnormal urine examination [9-12]. Proteinuria remains the most commonly observed anomaly in patients with LN [9,13-14]. Other common clinical presentations include but are not limited to, hypertension, microscopic hematuria with or without red cell casts, acute or chronic kidney disease, and nephrotic syndrome [9-14]. In SLE patients who develop active urinary sediment with persistent hematuria (five or more red blood cells per high-power field, mostly dysmorphic) and/or cellular casts, proteinuria, and/or elevated serum creatinine or decrease in estimated glomerular filtration rate, suspect LN [10,11]. All patients with lupus should be monitored regularly for LN.

The diagnosis of suspected LN is ideally confirmed with a kidney biopsy [10,11,15]. Kidney biopsy not only plays a key role in clinching the diagnosis of LN but also excludes other causes of kidney injury, determines the histopathologic subtype of LN, and ascertains disease chronicity and activity in LN [15]. Evaluating all these parameters is important as they can influence the treatment of patients with LN. We performed a kidney biopsy on our patient because he had nephrotic range proteinuria together with increasing serum creatinine levels, which could not be attributable to other pathology. These indications were consistent with some of the indications for kidney biopsy recommended by the joint European Alliance of Associations for Rheumatology/European Renal Association-European Dialysis and Transplant Association (EULAR/ERA-EDTA) guidelines [16].

Lupus nephritis can have different histopathological descriptions, but one major description is the full-house immunofluorescence pattern. This is characterized by glomerular deposits that stain predominantly for IgG and contain co-deposits of Immunoglobulin G, A, M (IgG, IgA, IgM), Complement 1q (C1q), and Complement 3 (C3) (3+) [17]. According to the ACR/EULAR 2019 Lupus Nephritis classification criteria, our patient had immune complex-mediated diffuse proliferative glomerulonephritis with full-house staining, which is consistent with one of the most common histopathological descriptions of LN. Infrequently, the "full house" immunofluorescence pattern is seen in patients with endocarditis, HIV, hepatitis C virus infection, a portosystemic shunt, and post-streptococcal glomerulonephritis [18-20]. However, the workup was negative for all these conditions; hence, a diagnosis of full house staining from LN was made.

According to the 2019 Update of the Joint European League Against Rheumatism and European Renal Association-European Dialysis and Transplant Association (EULAR/ERA-EDTA) recommendations for the management of lupus nephritis, the goal of therapy is complete response (proteinuria <0.5-0.7 g/24 hours with near-normal glomerular filtration rate) by 12 months, although this can be extended in patients with baseline nephrotic-range proteinuria. [21]. In active proliferative LN, induction treatment with mycophenolate mofetil (2-3 g/day or mycophenolic acid at an equivalent dose) or low-dose intravenous cyclophosphamide (500 mg × 6 biweekly doses), both combined with glucocorticoids (pulses of intravenous methylprednisolone 250-1000 mg daily for three days, then oral prednisone 0.3-0.5 mg/kg/day), is recommended [21]. Long-term maintenance therapy with mycophenolate or azathioprine with no or low-dose (<7.5 mg/day, ideally <5 mg/day) glucocorticoids should be continued after the induction treatment [21]. In pure membranous LN with nephrotic-range proteinuria or proteinuria >1 g/24 hours despite renin-angiotensin-aldosterone blockade, mycophenolate in combination with glucocorticoids is preferred [21]. Our patient was prescribed both prednisolone and mycophenolate mofetil and kidney function stabilized.

## Conclusions

This case underscores the diagnostic challenges in patients with multiple comorbidities, the importance of a multidisciplinary approach, and the complexity of managing glomerulonephritis in diabetic patients, highlighting the need for a thorough work-up, including kidney biopsy, to differentiate between autoimmune, infectious, and diabetic kidney disease; idiopathic membranoproliferative glomerulonephritis (MPGN); and seronegative lupus nephritis, which is a very rare case.
